# Involvement of the Wnt pathway in BVDV cytopathogenic strain replication in primary bovine cells

**DOI:** 10.1186/s12985-022-01863-6

**Published:** 2022-08-19

**Authors:** Rémi La Polla, Marie-Claire Testard, Océane Garcia, Abdelghafar Goumaidi, Catherine Legras-Lachuer, Blandine de Saint-Vis

**Affiliations:** 1 Site Lyon porte des Alpes, Boehringer Ingelheim Animal Health, 813 cours du 3eme Millenaire, 69800 Saint Priest, France; 2 Laboratoire d’Écologie Microbienne - UMR 5557, Université Claude Bernard Lyon 1, 43 Boulevard du 11 Novembre 1918, 69622 Villeurbanne Cedex, France; 3 Faculté de Médecine et de Pharmacie, Viroscan3D, 8 Avenue Rockefeller, 69373 Lyon, France

**Keywords:** BVDV-1, Cytopathogenic, Transcriptomics, Wnt pathway, Viral replication

## Abstract

**Background:**

*Bovine viral diarrhea virus 1* (*BVDV-1*) of the pestivirus genus is an economically crippling virus in the cattle industry; this positive RNA virus causes mucosal disease resulting in reproductive losses and other disease syndromes. The pathogenesis mechanism of the disease caused by BVDV infection is not well understood; for a better understanding of in vivo host BVDV-1 interactions, we conducted a transcriptomic study of infected cells at different times post-infection.

**Methods:**

We compared the permissiveness and cellular response of a BVDV-1 cytopathogenic strain on Madin-Darby Bovine Kidney cells (MDBK) and bovine lung primary cells, a model closer to in vivo infection. Then a RNAseq analysis was realized on the infected bovine lung primary cells, at 10 hpi and 30 hpi (hours post-infection), to identify transcriptomic signatures.

**Results:**

RNAseq analysis on BVDV-1 infected bovine primary cells showed 2,759 and 5,376 differentially expressed genes at respectively 10 hpi and 30 hpi with an absolute Fold Change  ≥ 2. Among the different pathways deregulated, data analysis revealed a deregulation of Wnt signaling pathway, a conserved process that play a critical role in embryogenesis, cellular proliferation, and differentiation as well as in viral responses against viruses such as *Influenza* or *Hepatitis C.* We demonstrated here that the deregulation of the Wnt/βcatenin signaling pathway plays a role in viral replication of BVDV cp strain. Interestingly, we showed that the inhibition of this Wnt pathway using two inhibitors, FZM1 and iCRT14, induced a delay in onset of the establishment of a cytopathic effect of primary cells.

**Conclusions:**

Thereby, this study highlighted a role of the Wnt signaling pathway in the BVDV-1 viral replication in bovine cells, suggesting an interesting option to explore as a new therapeutic target.

## Introduction

*Bovine viral diarrhea virus* (*BVDV*), belonging to the *Pestivirus* genus of the *Flaviviridae* family, is causative organism of bovine viral diarrhea and mucosal disease, a disease that causes reproductive losses and a range of other syndromes in cattle like congenital malformation and immunosuppression [1]. The virus is contracted from contact between infected cattle through saliva excretion or other contacts with fluids and, following infection, BVDV can infect almost every organ system such as gastrointestinal or respiratory tracts but appears to be dependent on *BVDV* species [2, 3]. BVDV is a positive-sense RNA virus of 12 kb with a 5’ and a 3’ untranslated region framing one open reading frame which will be translated into only one polyprotein then 12 proteins [1]. Two biotypes of *BVDV* are recognized: cytopathogenic (cp) or non-cytopathogenic (ncp), a criterion defined by the strain’s capacity to induce a cytopathic effect (CPE) in cell culture. In cattle, both biotypes are involved in mucosal disease, a disease emerging in an animal Persistently Infected (PI) by a ncp strain which is superinfected by a cp strain [3].

As per most viruses, BVDV uses and deregulates the host components and cellular pathways at different steps of the life cycle, such as antiviral pathways, inflammatory response and apoptosis. Several studies report also a deregulation of the Wnt canonical pathway, a conserved process playing a critical role in embryogenesis, cellular proliferation and differentiation,

This Wnt pathway has been recently reported as playing a role in host responses to infection including viral infection [4] both for DNA viruses such as Herpes Simplex virus [5], Epstein Bar virus [6], Cytomegalovirus [7], Hepatitis virus type B (HBV) and RNA viruses with Human Immunodeficient virus [8, 9], Hepatitis virus type C [10], Dengue virus [11], Rift Valley Fever virus [12], Sendai virus [13] or *Influenza* [14].

Wnt signaling pathway is triggered by WNT ligands binding to Frizzled (FZD) 7-transmembrane domain receptor, low-density lipoprotein-relation protein (LRP5, LRP6) receptors on the cell surface as well as receptor tyrosine kinases ROR and RYK. Depending on this receptor context, WNT ligands then activate distinct intracellular pathways, which can be grouped into β-catenin dependent and β-catenin independent signaling events. Briefly, in the β-catenin dependent pathway, binding of WNT to FDZ receptor and LRP co-receptor promotes the destruction complex stabilization of cytoplasmic β-catenin that then is translocated to nucleus and enables to active transcription factors of the TCF/LEF family. The Wnt response genes are regulated, such as the MYC and PPAR transcription factors involved in cell proliferation and lipid metabolism respectively, or the Cyclin D gene involved in cell cycle [15–17]. Among these genes, the receptors FZD and LRP, are generally downregulated while antagonist *DKK* are overexpressed to establish a feedback control of the Wnt pathway [18–20].

In the independent β-catenin signaling pathways, Wnt/Jnk signaling in conjunction with ROR or RYK co-receptors activates via RAC1 and RHOA the transcription factors of the JUN family, whereas Wnt/Ca^2+^ signaling activates the transcription factors of the NF-AT family [21].

A transcriptomic study of in vitro BVDV-1 infected cells showed an activation of the Wnt/β-catenin signaling pathway at a transcriptional level [22]. However, this study was performed on MDBK cells, an epithelial cell line derived from bovine kidney and did not investigate the effect of the Wnt pathway inhibition in viral replication.

In the present study, to be closer to an in vivo infection, we have chosen to work with primary pulmonary cells from bovine lung, an in vivo target cell type of BVDV [2, 23]. First, a preliminary study was carried out on MDBK and primary cells to evaluate the permissiveness and the cellular response after BVDV-1 NADL infection. Then, we performed by RNAseq, at 10 hpi and 30 hpi (hours post-infection), a transcriptomic analysis of lung bovine cells infected with the cytopathogenic BVDV-1 NADL. A complementary study has showed the activation of the Wnt pathway after infection of primary cells with cytopathogenic strain compared to non-cytopathogenic strain [24]. Here, data analysis confirmed a significant activation of the Wnt signaling pathway and demonstrated an implication of the β-catenin dependent pathway. To investigate the role of this Wnt pathway by BVDV-1 we used two inhibitors, iCRT14 and FZM1, and revealed both a reduction of viral production and a delay in apparition of CPE, suggesting a key role of the Wnt/β-catenin signaling pathway in the viral replication and pathogenicity of the BVDV-1 virus in mucosal disease.

## Methods

### Cells and viruses

Madin-Darby Bovine Kidney cells (MDBK) were cultured in a rich glucose medium (BI medium) supplemented with 5% of fetal bovine serum (FBS) in a 150 cm^2^ flask at 37 °C with 5% CO_2_.

Several bovine lungs were collected from healthy animals (Centre de Recherche de Saint Vulbas, BI France). Several pieces were placed in a GentleMACS tubes (Miltenyi Biotec) for grinding. The lysates were then filtered on a 40 µm filter and centrifugated at 700 g during 10 min. The cell pellets were resuspended in a rich glucose medium (BI medium) supplemented with 10% of FBS. The obtained bovine lung primary cells (BPC) were pooled from 4 different lungs then cultured and conserved in nitrogen liquid with 10% DMSO.

The strain BVDV-1 NADL cp (AJ133738) were amplified internally on bovine cells. Viral titers in fluorescent antibodies infectious dose 50% (FAID50/mL) were determined on BPC or MDBK after 3 days with a pool of anti-Pestivirus monoclonal antibodies (BI collection) and Goat anti-Mouse IgG Secondary Antibody, Alexa Fluor 488 (Invitrogen). The CPE were observed on BPC or MDBK with ZOE Fluorescent Cell Imager (Biorad).

### Kinetics protocol

BPC and MDBK were cultured for 3 days at 37 °C with 5% CO2 in 24 well plates at a starting density of 3 × 10^5^ cells/well. After 3 days, cells were inoculated with BVDV-1 NADL cp at a multiplicity of infection (MOI) of 1 or non-infected. The infection was done in triplicates.

To study cellular responses, cell cultures were harvested for RNA extraction at 0, 6-, 15-, 24-, and 30-h post-infection. At each time, cell lysates were harvested with RLT buffer (Qiagen), and total RNA was extracted using RNeasy Plus Mini Kit (Qiagen) and stored at − 70 °C.

### Droplet digital PCR

Collected cells were screened for the detection of the E2 region of BVDV-1 and 7 genes involved in antiviral response. The PCR primers used in this study were designed with UGENE software (Table 1) [25]. Droplet digital PCR was performed on 5µL of RNA extract with One-step RT-ddPCR Advanced Kit for Probes, AutoDG and QX200 system (Biorad). Reaction mixtures were incubated for 60 min at 50 °C, 10 min at 95 °C, followed by 40 cycles of 30 s at 95 °C, 1 min at 60 °C, and finally 10 min at 98 °C. For each sample, housekeeping gene TBP (Tata Binding Protein) was used to normalize.

**Table 1:** Primers and probes sequences

Target (Accession number)	Foward(5'- > 3')	Reverse (5'- > 3')	Probe (5'- > 3')
BVDV NADL (E2 region)	CCAAAGATGCACGAGAGAAACC	CTCTTGCTTTCGCCCATCAA	TGCATACAAGAGCCTTGCCGACCAGTGT
TBP (NM0010745742)	GCGTTTTGCTGCTGTAATCA	GGAACTTCACATCACAGCTC	CGCACCACTGCACTGATATTCAGTTCT
TLR2 (KT601038)	GTGCTTTGTGGACAGCATGG	TTGGACAGGTCAAGGCTTTTCA	CAGGCTTCTTCTCTGTCTTGTGACCC
TLR3 (AJ812026)	AAAGGGACTTTGAGGCAGGT	GATTTAAACATYCCTCTTCG	ATGCAGTTCAGCAAGCTATTGAACAA
NFkB (NM_001076409)	CTGGAAGCACGAATGACAGA	AACATGAGCCGTACCACGCT	CTATAATCCCGGACTTTT GGT GC A
IL6 (EU276071)	CAAAATCTCTGCAATGAGAAAGG	GCTTCAGGATCTGGATCAGTG	GATGCTTCCAATCTGGGTTCAATC
IL8 (AF232704)	ATGACTTCCAAGCTGGCTGTT	ACAACCTTCTGCACCCACTTT	CATTCCACACCTTTCCACCCCAAATTTATC
Mxl (AF047692)	TTCCACCTGAAGAAGGGCTA	GCCATACTTCTGTAACTCCTCTGTT	ATCCCCTGCCTGGCAGAAAGACT
ISG15 (Ml 74,366)	TGCAGAACTGC ATCTC CATC	CATGAACACGGTGCACCCCT	AGACCAGTTCTGGCTGTCTTTTGAAG

### Transcriptomic study

Primary cells were cultured for 3 days at 37 °C in 24 well plates and at a starting density of 3 × 10^5^ cells/well in triplicate. The medium used is a rich glucose medium (BI) supplemented with 5% of FBS without anti-Pestivirus antibodies. Then, the cells were inoculated at a MOI of 1 with BVDV-1 NADL and harvested at 0–10–30 h post-infection. Total RNA was extracted from cells with RNeasy Plus Mini kit (Qiagen), quantified using the Quantifluor RNA system (Promega) and qualified using a Bioanalyzer 2100 (Agilent). The library was prepared using the NextFlex Rapid Directional RNAseq V2.0 (Perkin Elmer), quantified and qualified using the Quantus Quantification kit (Promega) and the Fragment Analyzer (AATI). Sequencing was performed on a NextSeq 500 Flow Cell High Output SR75 instrument (Illumina) with 12 samples per flow cell (corresponding to 46 million reads/sample).

### Differential expression analysis

Trimming step was performed with CutAdapt software and trimmed reads were mapped using kallisto v0.44.0 against Bovine transcriptome (ARS-UCD1.2 from Ensembl). A second analysis was performed with a reads subset against the bovine genome using Tophat v2.1.1, PicardTools v2.1.0 and FeatureCount v1.5.0 software. Then, normalization was performed with DESeq2 R packages v1.26.0 and differential expression was analyzed by Ingenuity Pathway Analyis (Qiagen). Threshold set to a minimum of 100 reads with an absolute FC ≥ 1.5 and a p-value ≤ 0.05.

### Wnt pathway inhibition

BPC were plated in 24 wells for 3 days at 37 °C at a starting density of 3 × 10^5^ cells/well in triplicate. Cells were inoculated at a MOI of 1 with BVDV-1 NADL and treated with 25 µM of iCRT14 (Sigma-SML0203) or 10 µM FZM1 (Merck-534358) at 1-, 2- or 3-h post-infection. The supernatants were harvested at 24 h post-infection and titred, in FAID50/mL, on MDBK with a pool of anti-Pestivirus monoclonal antibodies (BI collection) and Goat anti-Mouse IgG Secondary Antibody, Alexa Fluor 488 (Invitrogen).

The CPE were observed on BPC under microscope in white light.

## Results

### Comparison of BVDV-1 NADL susceptibility and viral replication of BPC cells in comparison to MDBK cells

**Fig. 1:**
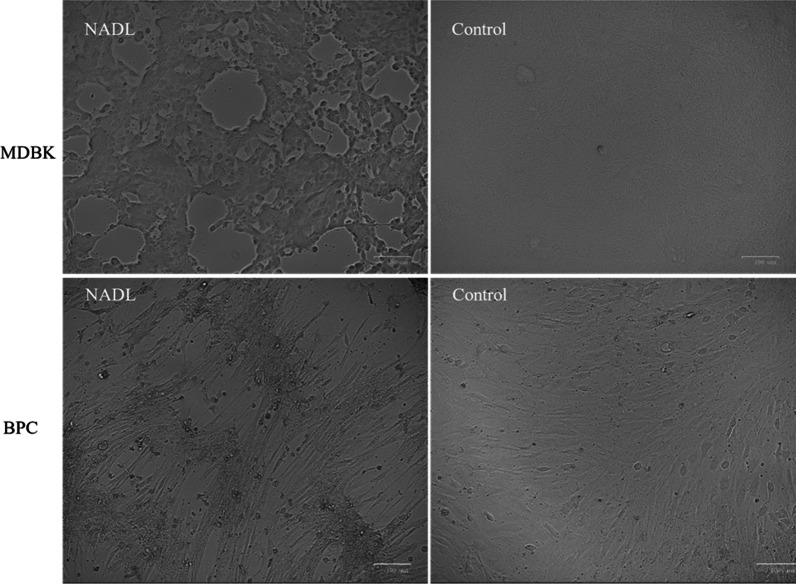
Microscopic observation of cytopathic effect on MDBK cells and BPC. Cells were infected at a MOI of 1 and observed with microscope in white light at 48 hpi

In order to compare the infection efficiency of BPC cells to BVDV-1 NADL, in comparison to the MDBK cells, 3 × 10^5^ cells were infected at an MOI of 1 by the NADL strain and then, observed at 8, 24, 48, 72 hpi, by microscopy. Results (Fig. 1) showed presence of CPE at similar levels for both infected cell types after 48 h only, confirming the permissiveness of BPC cells for the NADL strain. At 72 h post infection the CPE is total for the two cell types.

**Fig. 2:**
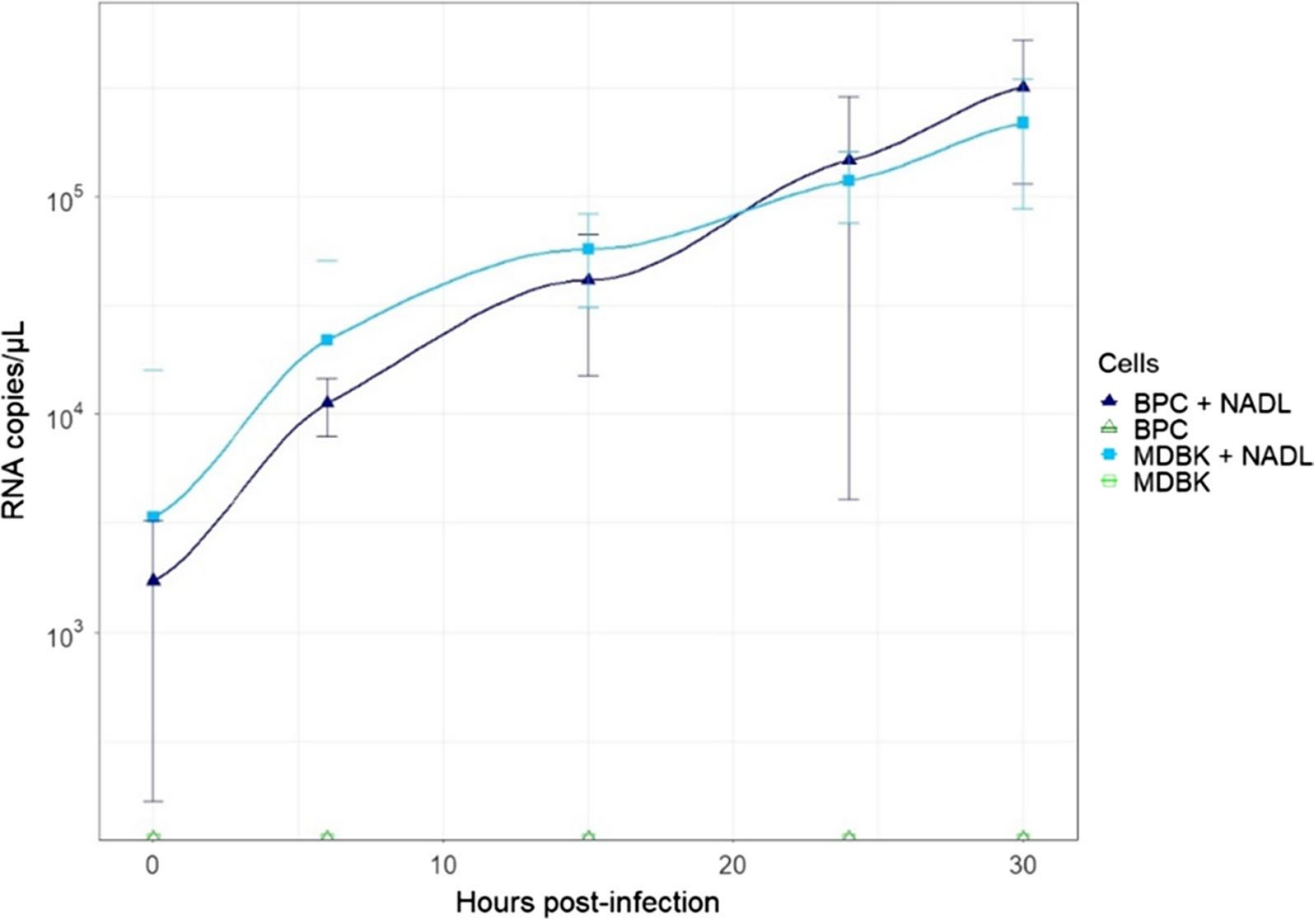
BVDV replication in bovine primary cells and MDBK. Viral RNA production follow by droplet digital PCR. The kinetics was done in triplicate. In dark blue corresponds viral RNA in BPC, in light blue viral RNA in MDBK and in green non infected BPC and MDBK cells. All quantifications were normalized with TBP (n = 3)

To evaluate viral replication, RNAs were extracted from both MDBK and BPC lysates, at different times post infection (0, 6, 15, 24, 30) and quantified by droplet PCR using specific primer of E2 region (See Method paragraph). Results (Fig. 2) showed a similar production of viral RNA in both MDBK and BPC.

### Comparison of cellular antiviral responses induced by NADL between MDBK and BPC cells

To assess antiviral cellular responses of BPC cells to NADL and compared them with BPC cells, we chose a panel of 7 genes involved in antiviral response *TLR2, TLR3, NFKB, IL6, IL8, ISG15* and *MX1,* and monitored by droplet digital PCR*,* their expression during time course of infection 0, 6, 15, 24, 30 h post-infection.

**Fig. 3:**
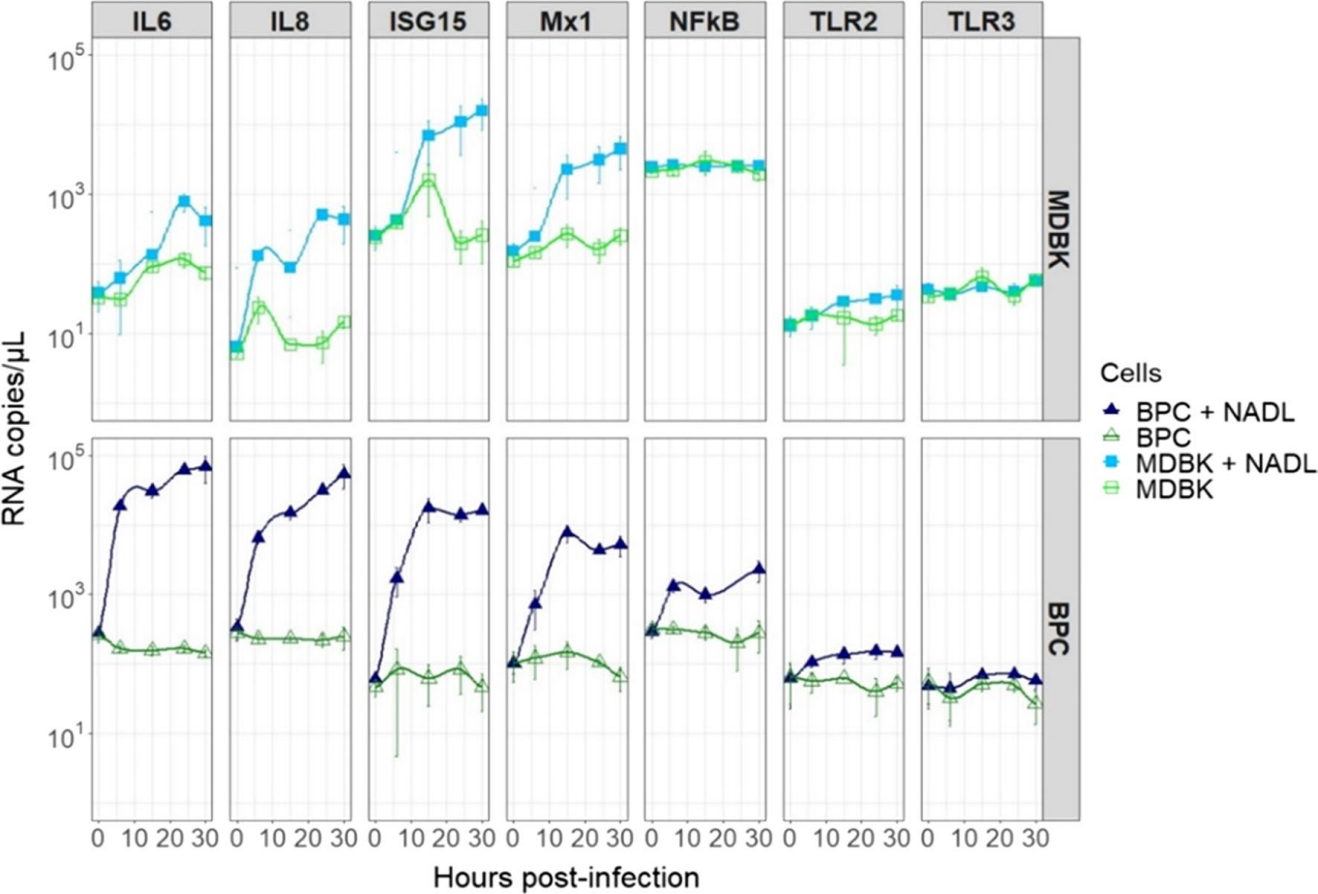
Cellular gene expression of BPC and MDBK followed by droplet digital PCR. The kinetics was done in triplicate. In dark blue corresponds BPC infected, in light blue MDBK infected and in green non infected BPC and MDBK cells. All quantifications were normalized with TBP (n = 3)

Results (Fig. 3) showed an overexpression of *IL-6, IL-8, ISG15, MX1* and *TLR2* genes, in infected MDBK and BPC cells, but an overexpression only in BPC cells, of the *NFKB* and *TLR3* (10^4.7^ in BPC infected vs. 10^2.6^ copies/µL in uninfected cells) genes suggesting a better antiviral response of the target BPC cells to NADL infection. This is supported by the levels of expression of *IL6* (10^5^ copies/µL for BPC vs 10^3^ copies/µL for MDBK) and *IL8* (10^4.7^ vs 10^2.6^ copies/µL) genes that were lower in infected MDBK compared to infected BPC.

### Analysis of the Wnt signaling pathway genes responses after BVDV-1 NADL infection

In order to better understand the host–pathogen interactions, the cellular gene expression induced by BVDV-1 was analyzed by transcriptomic analysis, using RNASeq. For this, total cellular RNA was extracted from infected BVDV-1 NADL (MOI 1) or non-infected cells, at 0, 10, and 30 hpi and then sequenced using Illumina NextSeq 500 technology.

**Fig. 4:**
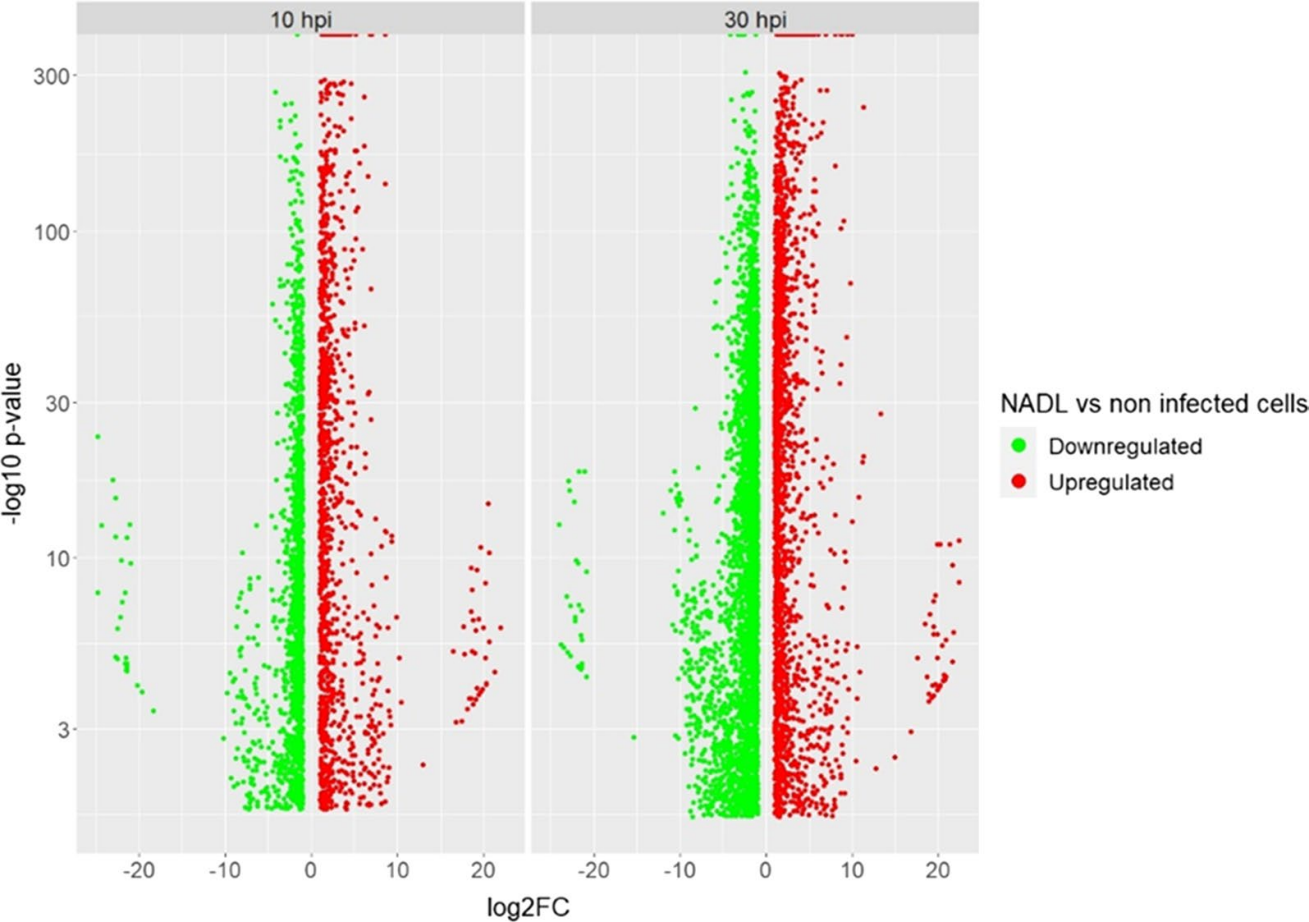
Homogeneous distribution of genes deregulation in infected BPC. Genes significantly downregulated are represented by green spots and genes significantly upregulated are represented by red spots

Data analysis revealed 2759 differentially expressed genes at 10 hpi and 5376 at 30 hpi with an absolute Fold Change (FC) ≥ 2 (*p*-value ≤ 0.05). As expected for this type of study, we observe a homogeneous distribution of upregulated and downregulated genes for both time points (Fig. 4).

To complete this global analysis, a focus using Ingenuity Pathway Analyis was performed on the Wnt signaling pathway identified previously by PANTHER database and activated after infection with the cytopathogenic biotype [24]. In response to viral infection, WNT ligand are involved in the positive or negative regulation of type I interferon. For example, WNT2B and WNT9B were identified as negative regulators of Sendai virus-inducted interferon-β; WNT5A as positive, and WNT7B, WNT9B as negative regulators of Influenza A replication; WNT2 and WNT3 as positive regulators of Dengue virus but their functions in the context of immune responses are incompletely understood and role of this Wnt pathway in BVDV infection has been yet never described.

**Fig. 5:**
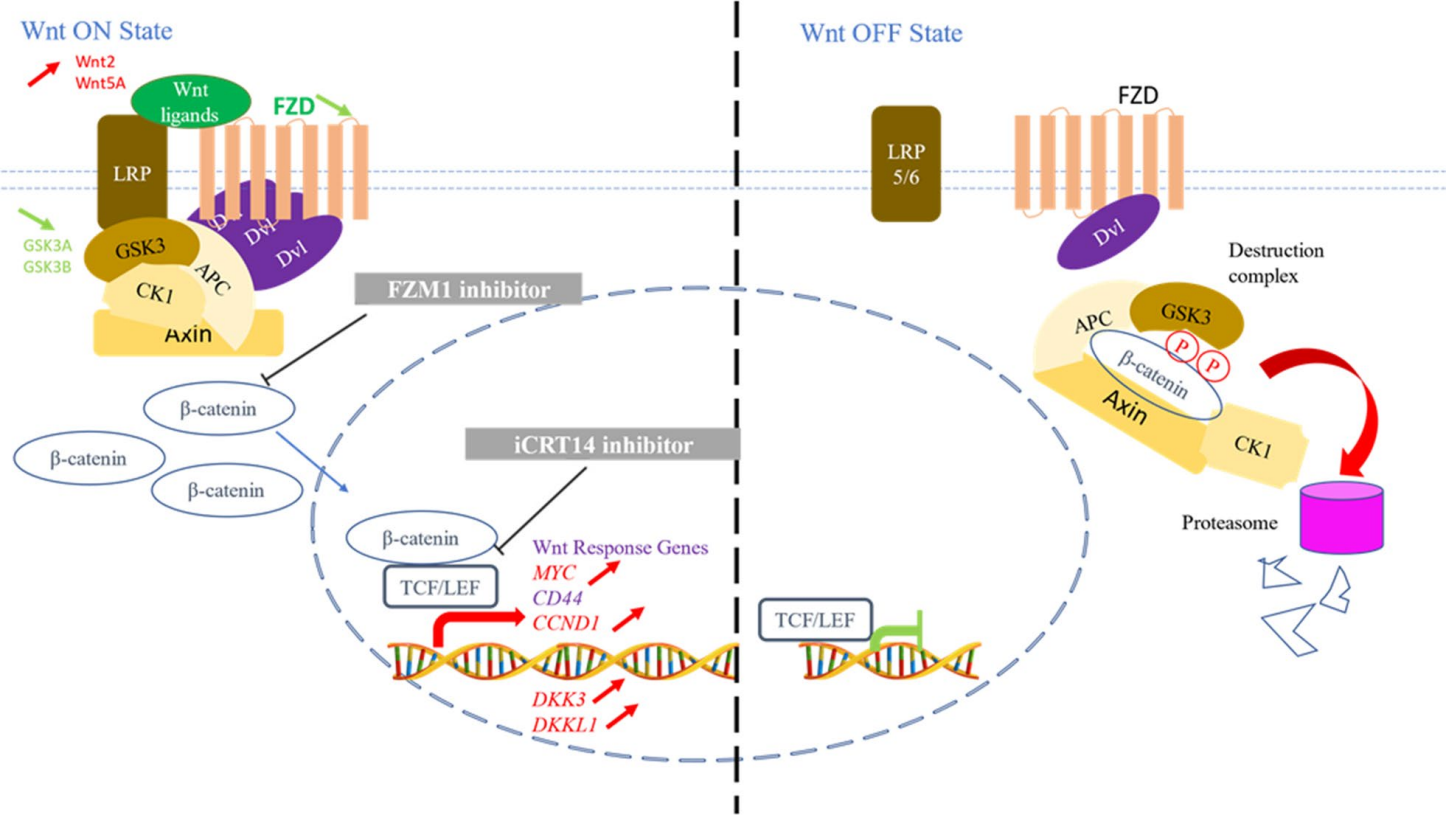
Schematic representation of the Wnt canonical pathway. The Wnt canonical pathway is activated after WNT ligand binding to receptors complex Frizzled-LRP5/6. This interaction allows the formation of a complex, the “signalosome”, that blocked β-catenin degradation leading to its accumulation in cytosol and translocation in nucleus. The β-catenin forms a transcription complex with the transcription factor TCF and LEF to activate genes expression. On the OFF state, β-catenin is continually degraded by the destruction complex which promotes β-catenin phosphorylation leading to its degradation by the proteasome. Some interesting genes identified in Table 2 are reported with arrows (red color for upregulated genes and green for downregulated genes)

We found a down expression of the receptors belonging to the Wnt pathway including *FZD 2, 4, 7, 8* genes (FC − 5.48, − 3.34, − 3.31 and − 3.03, respectively at 30 hpi), a down expression of *LRP1* and* 6* (− 13.25 and − 1.88 at 30 hpi), involved in the feedback control, and an overexpression of *DKK 3* and *DKKL1* genes (2.81 and 3.30 at 30 hpi), antagonist of the WNT ligands, confirming the involvement of the Wnt pathway (Table 2) (Fig. 5).

**Table 2:**
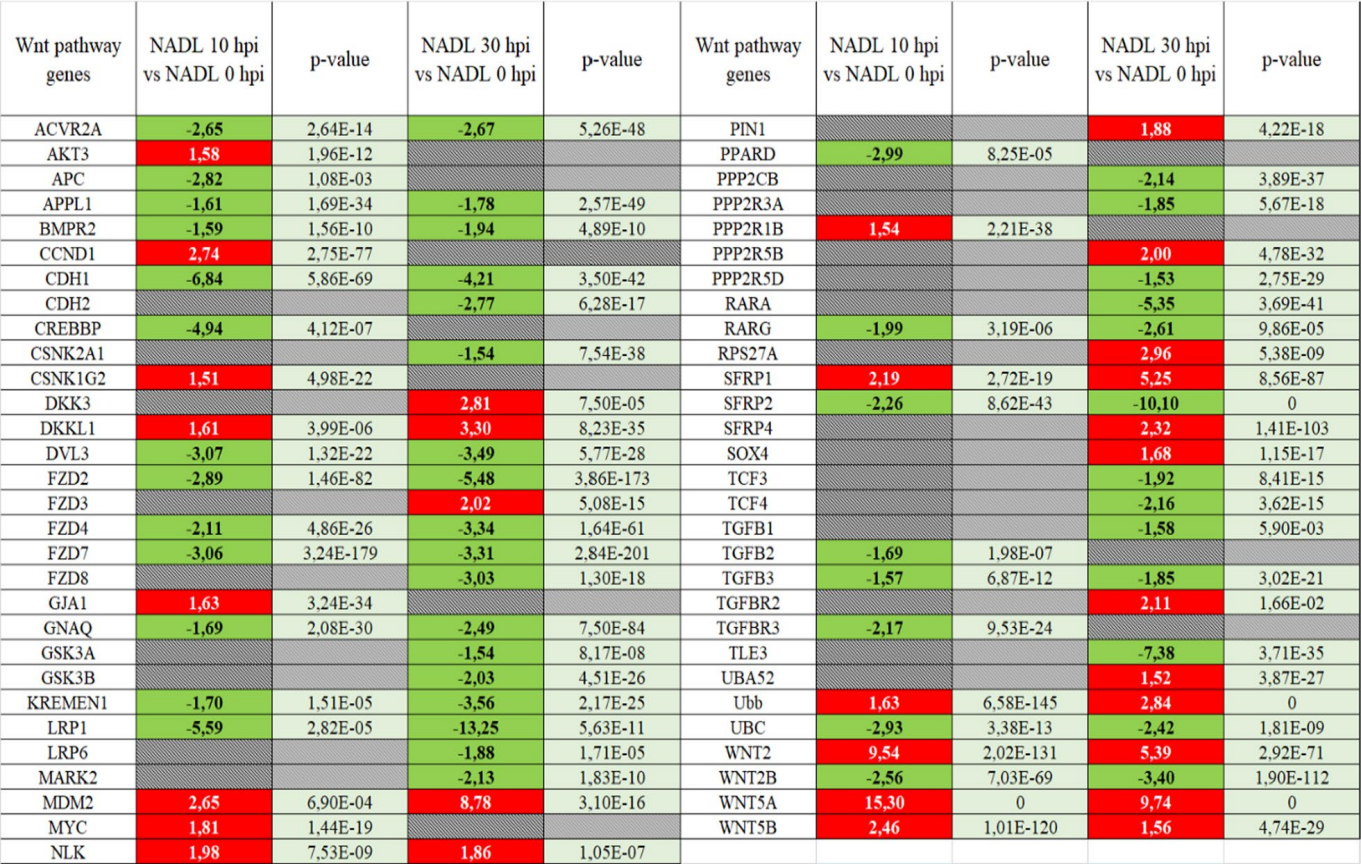
Transcripts fold change of genes involved in Wnt signaling pathway

Wnt signaling pathway is studied after cell infection by NADL at 10 hpi or 30 hpi versus 0 hpi. The genes upregulated are colored in red and the genes downregulated are colored in green. A threshold of 100 for reads counts and ≥ 1.5 for fold change is set.

Moreover, we found a dysregulation of gene expression involved the dependent β-catenin signaling including an overexpression of *MYC* (1.81) and *CCND1* (2.74), *Wnt2* (9.54/5.39) and *Wnt5A* (15.30/9.74) ligands, a downexpression of *GSK3A* (− 1.54) and *GSK3B* (− 2.03) involved in the ß-Catenin degradation (Table 2).

### Analysis of Wnt signaling pathway inhibition on BVDV replication

In order to understand the role of this Wnt/ß-catenin signaling pathway in BVDV-1 infection, an inhibition of this pathway was performed in infected cells, using two inhibitors of this pathway: the iCRT14 inhibitor that inhibits the of β-catenin-TCF interaction and the FZM1 inhibitor that promotes the phosphorylation of the β-catenin and its degradation. BPC cells were infected at a MOI of 1 with BVDV-1 NADL cp strain and 25 µM of iCRT14 and 10 µM of FZM1 was added at 1-, 2- or 3-h post-infection. Cell viability in the different conditions was evaluated using a Vi-Cell cell counter and viability analyzer and no impact was observed (data not shown).

**Fig. 6:**
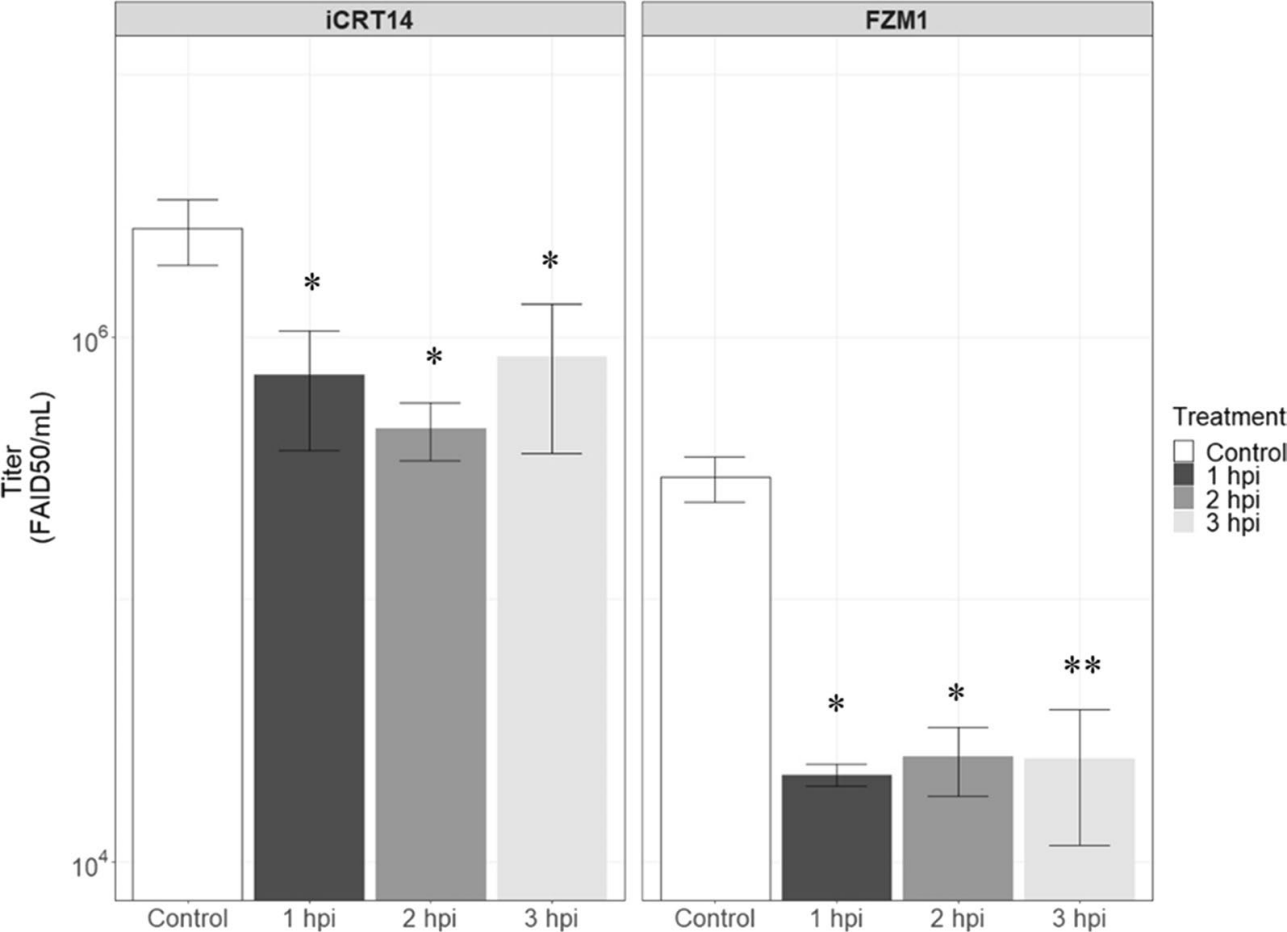
BVDV NADL viral production on BPC treated with iCRT14. BPC were plated in 24 wells for 3 days then infected with BVDV NADL cp strain at a MOI of 1. FZM1 or iCRT14 was added in culture at 1-, 2- or 3-hours post-infection and cells were harvested at 24 hpi. The viral protein is labeled with a pool of anti-Pestivirus antibodies then revealed with Alexa488 (green). Titration was performed on MDBK in duplicate for each replicate (n=3). Student test p-value * <0.05 and ** <0.01

**Fig. 7:**
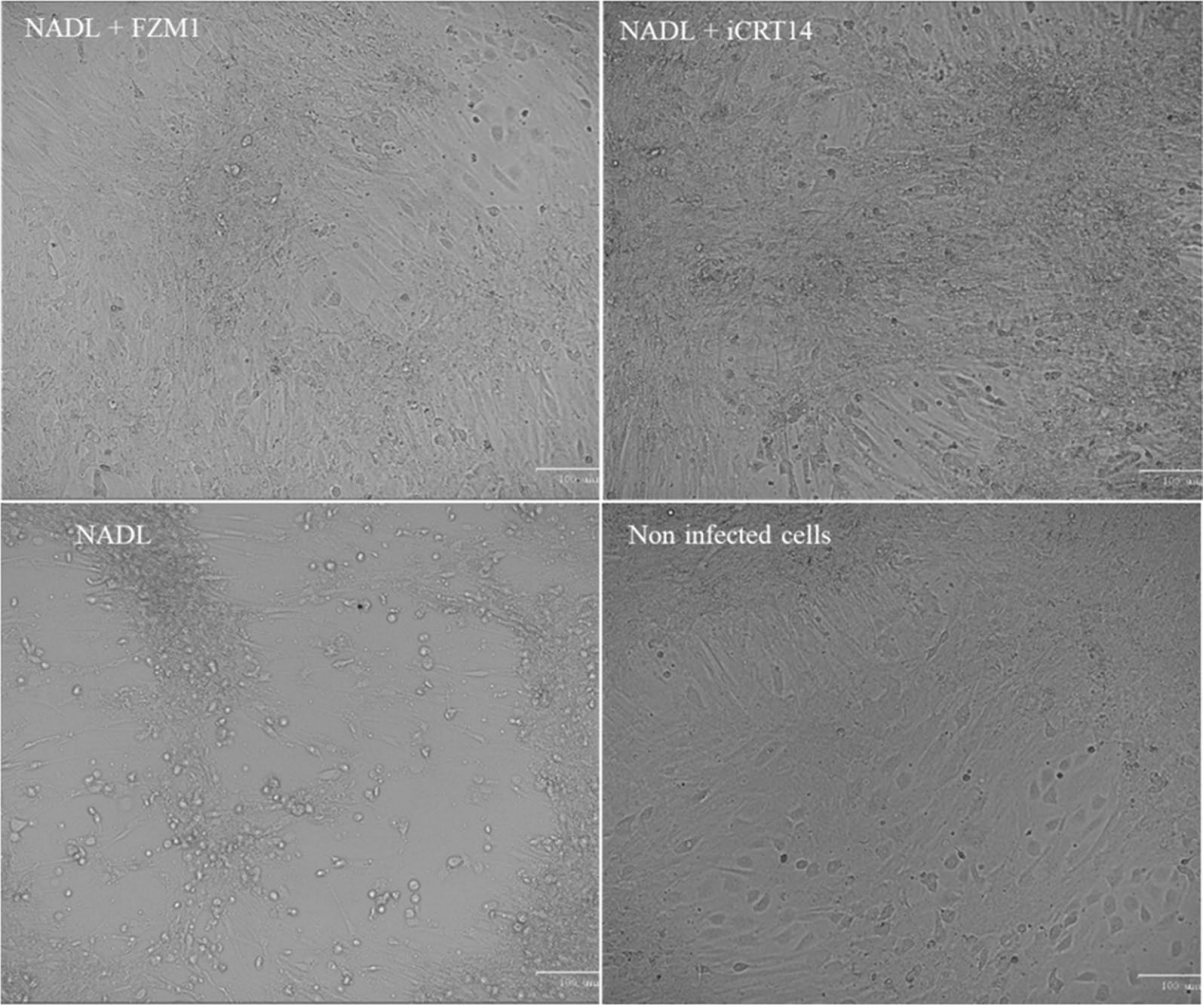
Microscopic observation of cytopathic effect on BPC. Cells were infected at a MOI of 1 and treated 1 hpi with inhibitor FZM1 or iCRT14 at 10 µM and 25 µM respectively. Cells were observed under microscope in white light at 48 hpi. Titration was performed on MDBK in duplicate for each replicate (n = 3). Student test *p*-value * < 0.05

Then, supernatants were collected from BPC infected cells, at 24 h post infection and then titrated on MDBK cells (Fig. 6). Moreover, BPC infected cells treated 1-h post-infection with inhibitors were observed by microscopy at 48 hpi to evaluate the presence of ECP. Results showed a fivefold diminution of virus titers when the iCRT14 inhibitor is add 2 h post infection (10^6.4^ FAID50/mL vs. 10^5.8^ FAID50/mL for the control) and a tenfold virus reduction with the FZM1 inhibitor (10^4.3^ FAID_50_/mL compared to 10^5.46^ FAID50/mL for the control) (Fig. 6). For microscopic observations we observed a complete absence of CPE with both FZM1 and iCRT14 inhibitors (Fig. 7).

In conclusion, altogether of our results demonstrated for the first time, the implication of the Wnt/ß-Catenin pathway in the viral replication of BVDV-1 NADL cp strain.

## Discussion

This present study has the objective to improve our knowledge about the cell response after infection by a cytopathogenic strain of *BVDV, in* primary bovine pulmonary cells, the in vivo target cells of the virus. Although previous work reports a transcriptomic analysis of cells infected with BVDV1 [2, 23] and under lighted the deregulation of different gene pathways including the Wnt pathway, this study was carried out on MDBK cells, an epithelial cell line derived from bovine kidney, and author did not investigate the role of the Wnt pathway in BVDV replication.

In a first time, we confirmed the interest of working on primary cells. Effectively, if we showed that the permissiveness and viral production of BPC are equivalent to those of MDBK cells, on the over hand, the antiviral response to BVDV is more important in BPC. Indeed, a targeted analysis of gene expression by droplet digital PCR, of 7 genes involved in antiviral response allowed us to show an overexpression of IL-6, IL-8, ISG15, MX1 and TLR2 genes in both MDBK cells and BPC but higher, in BPC cells (up to 100-fold for the IL6 and IL8 genes) and an overexpression of the NFKB and TLR3 genes only in BPC cells.

A previous study has demonstrated the activation of the Wnt pathway depending on the biotype of BVDV [24]. In this study RNAseq analysis confirmed, in BVDV-1 NADL infected BPC cells, an activation of the Wnt/β-catenin pathway with an overexpression of genes coding for WNT ligands (Wnt2 and Wnt5A) and Wnt response genes (MYC, CCND1, DKK3, DKKL1), down regulation of FZD and LRP receptor genes (Fig. 7). The involvement of the Wnt pathway was confirmed using iCRT14 and FZM1 inhibitors, that induced a reduction of viral production and a delay in apparition of CPE, suggesting a key role of the Wnt/β-catenin signaling pathway in the viral replication and production. WNT network in response to infections has driven significant interest. WNT ligands (and other ligands for WNT receptors) are known to contribute to the host control in peculiar, in polarization of the antigen-presenting cells (APCs), orchestrating phagocytosis, and inflammatory cytokine responses; they are also involved in viral infection by both DNA and RNA viruses, including as example Hepatitis C [10], HIV [8, 9], Influenza [14], and Dengue RNA viruses [11].

For HCV, a flavivirus closer to Pestivirus, Liu et al. have shown that the HCV Core protein activates the β-catenin/Tcf-4-dependent pathway and increases active β-catenin expression and upregulates gene expression of canonical WNT ligands and Frizzled receptors [26]. It has been also reported that the HCV NS5A protein activates β-catenin signaling cascades (Street) and that the interaction of NS5 with β-catenin leads to the stabilization of the cellular protein in cytoplasm [10, 27].

Concerning bovine viruses, it has been reported that bovine herpesvirus (HSV)1 stimulates the Wnt/b-catenin pathway [28] and that the iCRT14 inhibitor reduces productive infection [29].

The correlation between the apoptosis process and mucosal disease lesions due to cp strain superinfection has been established previously in BVDV [23]. Mucosal disease is triggered following the superinfection of a PI animal by a cp strain and is always fatal in cattle. So, it could be of interest to study the effect of the Wnt pathway inhibition on an in vivo model presenting mucosal disease. Understanding the functional nature and temporal regulation of WNT responses in the host response to infection to BVDV, is essential for identifying therapeutic opportunities.

## Conclusion

The pathogenesis mechanism of mucosal disease caused by BVDV infection is not well understood. Herein, through the identification of the involvement of the Wnt/β catenin pathway, we identified two specific inhibitors that would allow to better understand the pathogeny of the disease, and that could be evaluated as potential therapeutics target to minimize the BVDV load in animal.

## Data Availability

The datasets analyzed during the current study are available from the corresponding author on reasonable request.

## Abbreviations

BPC: Bovine lung primary cells
BVDV: Bovine viral diarrhea virus
CPE: Cytopathic effects
MDBK: Madin-Darby bovine kidney cells
MOI: Multiplicity of infection
PI: Persistently infected
hpi: Hours post-infection
